# Structure of Investor Networks and Financial Crises

**DOI:** 10.3390/e23040381

**Published:** 2021-03-24

**Authors:** Kęstutis Baltakys, Hung Le Viet, Juho Kanniainen

**Affiliations:** Computing Sciences, Tampere University, 33720 Tampere, Finland; hung.le@tuni.fi (H.L.V.); juho.kanniainen@tuni.fi (J.K.)

**Keywords:** investor networks, financial crisis, complex networks, network theory, network topology, stock markets

## Abstract

In this paper, we ask whether the structure of investor networks, estimated using shareholder registration data, is abnormal during a financial crises. We answer this question by analyzing the structure of investor networks through several most prominent global network features. The networks are estimated from data on marketplace transactions of all publicly traded securities executed in the Helsinki Stock Exchange by Finnish stock shareholders between 1995 and 2016. We observe that most of the feature distributions were abnormal during the 2008–2009 financial crisis, with statistical significance. This paper provides evidence that the financial crisis was associated with a structural change in investors’ trade time synchronization. This indicates that the way how investors use their private information channels changes depending on the market conditions.

## 1. Introduction

Market dynamics and investor behavior are inseparable: The state of markets can affect investor co-behavior in certain ways, and, on the other hand, investor behavior ultimately drives the price dynamics in stock markets. The financial literature provides strong evidence on overreactions in stock markets, even among professional analysts [1,2,3]. According to Shiller [4], “bubbles are essentially subtle social-psychological phenomena”. From this point of view, it is crucial to understand the extent to which investors’ co-behavior and mutual connections, and thus the use of private information channels, vary over different market conditions. Particularly, the question is if the ways in which investors mutually share and use private information differ across different market conditions.

Intuitively, investors may follow different peers in bull versus bear markets. In addition, it is possible that investors receive, perceive, and utilize received private information differently under different market circumstances. Moreover, investors may share information they possess with their fellow investors differently under different conditions. In all of these cases, investor networks estimated from shareholder registration data, which serve as proxies for information networks [5], should show different properties under different market conditions.

In this paper, we address the question of if and how investor networks change around financial crises. Our methodological framework comes from complex network theory, which has been successfully applied to model investors’ behavior and interactions in stock and FX markets. There are various estimation techniques used with rich sets of shareholder registration data [5,6,7,8,9,10,11,12,13,14,15]. The research on investor networks sheds light on investor’s trade timing synchronization, which is closely related to herding behavior. On the other hand, according to Ozsoylev et al. [5], an investor network is a valid proxy for an information network, describing private information channels between investors see also [11]. From this point of view, network techniques not only describe investors’ co-behavior but also identifies the mutual private information channels of the traders.

There are considerably many papers analyzing topological changes of economic and financial networks, especially of interbank and bank-asset networks [16,17,18,19,20] (for a methodological review on the statistical detection of structural patterns in real-world networks, see [21]). At the same time, to our best knowledge, this is the first paper that statistically investigates whether investor network topology changes across market conditions, which is the main contribution of this paper. Our question is motivated by the existing empirical evidence that investors are clustered and that the clusters of investors change over time [6,10,12]. Indeed, the literature has barely addressed how investor networks’ structure reflects market conditions. In this regard, one welcomed exception is [14], which is one of the most closely related articles to this one. It investigates the dynamics of the investor spanning trees constructed from their trading correlations in Nokia stock around the dot-com bubble (January 1998 to December 2002). The paper observes changes in the mean weights of the spanning tree links. However, it provides a pure exploratory analysis without statistical tests, and only single security is analyzed.

We conduct statistical tests with an extensive shareholder data set containing informaiton about transactions in hundreds of securities, particularly focusing our attention around the financial crisis of 2008–2009. We analyze the structure of annual investor networks inferred using all marketplace transactions of all securities executed in the Helsinki Stock Exchange by all stockholders between 1995 and 2016. These 22 annual periods represent a wide range of different market conditions. In this paper, we analyze investor networks for the investor categories instead of individual investors. Similarly to [9], we categorize investors into 122 groups based on their economic and social attributes. In this regard, households are categorized based on their age, postal code, gender, while institutions and corporations are based on the sector codes, and postal code. There are multiple advantages to using investor categories. First, the network size is smaller, which allows us to employ structural measures that would not be suitable to analyze in large networks with thousands of individual investor nodes that trade in the entire stock market. Second, the size of the network remains the same when using investor categories instead of individual investors that can enter and leave the stock market. Third, the issue of the sparse investor trading observations is eliminated by combining trades of multiple investors into categories. Moreover, the advantage of using investor categories over individual investors lies in the ability to make socio-economic interpretations about the behavior of (investor) nodes of the networks.

By leveraging the hypergeometric test [6,7] we infer 22 annual investor category networks, and estimate a set of topological features for each one of them. After this, we conduct a statistical analysis to compare the distribution of network features for all pairs of periods. We find that investor networks during the 2008–2009 financial crisis period have statistically different empirical network feature distributions compared to other periods. This indicates that investors behaved abnormally at this time. Furthermore, their co-behavior was different.

This paper contributes to the literature by proposing and validating techniques for comparing investor networks. Numerous network comparison techniques have been used and proven successful in various other scientific disciplines [22]. However, in particular, investor networks may have different characteristics, requiring tailored methods for comparing them. For example, investor networks are inherently dynamic as they depend on the investor executed transactions in the network estimation period, i.e., its structure can vary over time. We want to test the key topological features here: Network density, global clustering, the size of the largest connected component, average path length, and global efficiency. Moreover, the topological indices suggested by [23,24] can be used to compare networks. In this regard, the Wiener index, defined as the sum of the shortest path lengths between all pairs of nodes, is used to capture the graph topology. We also test a series of global network features that leverage the concept of centrality [25,26]. For example, Di Cerbo and Taylor [27] recently adopted the degree-based network-level centrality measure to investigate the dynamics of the stock correlation networks, which we also use, among other features.

## 2. Materials and Methods

### 2.1. Data Set and Network Inference

The data used in this study come from the central register of shareholdings for Finnish stocks from the Finnish central depository provided by Euroclear Finland. Detailed descriptions of the data set can be found in [6,9,12,14,28,29]. Our sample data consist of all marketplace transactions executed in the Helsinki Stock Exchange by all stock shareholders from 1 January 1995 to 31 December 2016. This includes more than 1.2 million investors and roughly 37,000 exchange-traded securities over the 22-year period.

Instead of focusing on individual investor networks, in this paper, we analyze the networks of investor categories. Following [9], we group investors into categories based on their economic and social attributes. Each investor in the data set is assigned to a sector category: Non-financial corporations, financial-insurance corporations, government institutions, non-profit institutions, EU institutions, non-EU institutions, and households. Households are further divided into five age groups: Under-aged (0,18), young (18,30), middle-aged (30,50), mature (50,64), and retired (64, +∞). Moreover, all of these groups are further divided into smaller groups based on postal codes of 11 geographical regions. Additionally, there is a separate category for foreign investors. In total, this makes N=122 distinct investor categories.

The majority of investors in the stock market belong to the household sector, e.g., 52,356 were households out of 58,000 investors who traded in 1995 or 266,175 out of 286,022 who traded in 2016. The second-largest group of investors is the non-financial corporations, e.g., 4565 non-financial corporations in 1995 traded in the Helsinki stock exchange, and in 2016 there were 12,473, see Table 1. Even though households are the most abundant investor group in the stock market, the majority of them are rather passive investors making infrequent trades in few securities [30]. This makes the estimation of investor networks for the whole population an unfeasible task. Instead, if the individual investors are grouped into categories based on their socioeconomic attributes, most of the categories have an ample basket of observations for the inter-category relationships to be established, see Table 2.

Next, to infer the annual investor category networks we follow the methodology used in [6,10]. First, for each security *k*, for each investor category *i* and each trading day *t* we calculate the net-scaled-volume as:

(1) $$v_{i,t,k}=\frac{B_{i,t,k}-S_{i,t,k}}{B_{i,t,k}+S_{i,t,k}},$$

where $B_{i,t,k}$ and $S_{i,t,k}$ respectively are the total purchased and sold volumes in some security *k* of investor category *i* on day *t* respectively. Based on the net-scaled volumes we assign one of two trading states–*b* (primarily buying, when $v_{i,t,k}>\theta$ with θ=0.05) or *s* (primarily selling, when $v_{i,t,k}<-\theta$ with θ=0.05).

Separately for each security, using the assigned trading states in a given year, we link two investor categories if both of them have been in the same trading state at least once during that year, thus creating annual security-specific networks. To validate the links of a given annual security network for each investor category pair, we perform a hypergeometric test to check whether we can reject the null hypothesis of random trading state co-occurrence. Here we look for the same trading state co-occurrences P∈{b,s}, i.e., we check if both investor categories have been primarily buying or selling on the same days. To calculate the associated *p*-value we calculate the number of days when investor categories *i* and *j* have been in a trading state *P* in total and in the intersection, $N_{i,k}^{P}$, $N_{j,k}^{P}$, and $N_{i,j,k}^{P}$ respectively. Then, if the total number of trading days in security *k* in a given year is defined as $T_{k}$, the probability of observing *X* co-occurrences among $T_{k}$ observations is defined by the hypergeometric distribution $H(X|T_{k},N_{i,k}^{P},N_{j,k}^{P})$ and corresponding *p*-value for a link between two investor categories *i* and *j*, defined as:

(2) $$p\left(N_{i,j,k}^{P}\right)=1-\sum_{X=0}^{N_{i,j,k}^{P}-1}H\left(X|T_{k},N_{i,k}^{P},N_{j,k}^{P}\right),$$

where

$$H\left(X|T_{k},N_{i,k}^{P},N_{j,k}^{P}\right)=\frac{\left(\;\;\begin{array}{c} N_{i,k}^{P}\\ X \end{array}\;\;\right)\left(\;\;\begin{array}{c} T_{k}-N_{i,k}^{P}\\ N_{j,k}^{P}-X \end{array}\;\;\right)}{\left(\;\;\begin{array}{c} T_{k}\\ N_{j,k}^{P} \end{array}\;\;\right)}.$$

The null hypothesis of the hypergeometric test in this context is that investor categories *i* and *j* time their transactions randomly and independently. That is, if we reject the null hypothesis with a relatively low *p*-value, then it is unlikely that the trade synchronization of two investor categories, observed from actual trading data, can be explained by randomness. In that case, we say that the two investor categories in question are connected in the network with statistical significance.

In the literature on financial networks, alternative estimation techniques have been used, including partial correlation [31,32], correlation threshold networks [33], and cross-correlation function (CCF)-based Granger causality to test spillover effects [20,34]. Different network inference methods can be combined with network filtering procedures such as the minimum spanning tree or the planar maximally filtered graph (PMFG) method [35,36,37], among others (for an extensive review of the inference methods on financial networks, see [38]). Moreover, there are entropy-based approaches introduced in [39,40]. In addition, numerical techniques, such as the conservative causal core network with bootstrapping [9] could be used. Overall, there are many alternative techniques, but in this paper, we focus on using the hypergeometric test for two important reasons: (i) It can be used with sparse data, and (ii) it is not sensitive to outliers. Moreover, to our best knowledge, the method introduced in [6] is the one of the most widely used methods to estimate investor networks (see, for example, [10,11,12,13]).

**Figure 1:**
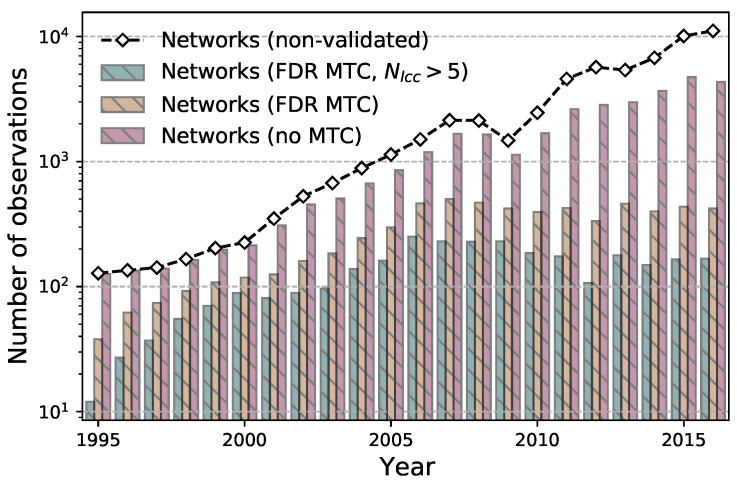
The number of security-specific networks in a given year with a different link validation: (i) Networks with at least one non-validated link (black curve with diamond-shaped markers), (ii) networks with at least one link with a *p*-value lower than 0.05 (red bars), (iii) networks with at least one link remaining after the false discovery rate (FDR) multiple test correction (MTC) was applied (yellow bars), and (iv) networks with more than five nodes in the largest connected component after the FDR MTC was applied (green bars). See also Table A1 in the Appendix A.

In this paper we have used the statistical significance α=0.05, which is further adjusted using the false discovery rate (FDR) multiple test correction [41], where the number of tests is fixed for each network and equal to $n_{\mathrm{tests}}=N\times \left(N-1\right)$. First, we sort the *p*-values of all $n_{\mathrm{tests}}$ statistical tests from the lowest to the largest (investor category pairs that are not linked are assigned a *p*-value of 1.)—$p_{1}\leq p_{2}\leq \ldots \leq p_{n_{\mathrm{tests}}}$. Then, we retain the links that satisfy $p_{i}<\alpha \cdot i/n_{\mathrm{tests}}$, where $i=1,\ldots ,n_{\mathrm{tests}}$. We then find the largest $l_{\max}$ such that $p_{l_{\max}}\leq \alpha \cdot l_{max}/n_{\mathrm{tests}}$ and select the links by rejecting the null hypothesis for the tests with $p_{1},p_{2},\ldots ,p_{l_{\max}}$. This procedure is done separately for the two types of trading states (*b*, *s*), after which security-specific annual networks are obtained by taking the union of the links over the buying and selling behavior networks. Finally, we keep only the networks with at least 5 nodes in the largest component for the network feature analysis. For the numbers of inferred networks see Figure 1 and Table A1 in the Appendix A.

Alternatively to FDR, other methods for multiple comparisons, such as Bonferroni correction, could be used. In comparison to Bonferroni, FDR, however, has some advantages. With Bonferroni, the adjusted threshold $\alpha /n_{\mathrm{tests}}$ is applied to all the links, being very conservative. It is a simultaneous test of a ‘universal’ null hypothesis against an omnibus alternative hypothesis [42]. For that reason, Bonferroni increases type II errors (false negative) [43], that is, actual links can be accidentally removed. Nevertheless, for the robustness check, we compare the results with both FDR and Bonferroni.

### 2.2. Network Features

In this paper, the investor networks are analyzed in light of the most prominent network features typically found in economic and financial network research [25]. The goal is to observe if the distributions of certain network features change over the years, with a particular focus on the crisis period in the years 2008 and 2009.

For all inferred networks, we calculate the set of investigated global network features. Those include—the number of links *L*, density ρ, and average degree 〈k〉 in the network:

(3) $$\begin{array}{cc} L & =\frac{1}{2}\sum_{i,j}^{N}A_{ij}, \end{array}$$

(4) $$\begin{array}{cc} \rho & =\frac{2L}{N(N-1)}, \end{array}$$

(5) $$\begin{array}{cc} \langle k\rangle & =\frac{1}{N}\sum_{i=1}^{N}k_{i}=\frac{1}{N}\sum_{i=1}^{N}\sum_{j=1}^{N}A_{ij}=\frac{2L}{N}, \end{array}$$

where *N* is the number of nodes in a network (N=122), $k_{i}$ is the degree of node *i*, and *A* is the adjacency matrix of an unweighted, undirected network, without self-loops. Here, the network density ρ is computed as the total number of links divided by all possible links in a given network. That is, density measures how many of all possible links exist in a given network. The average degree of an investor category 〈k〉 describes the average number of connections it has to other categories in the network. Since the network density and average degree are linearly dependent on the number of links, the results will be reported only for the latest. These basic features can be used to measure the overall connectedness in the network. In terms of investor networks, they measure the level of synchronization in investor trade timing associated with their herding behavior.

Additionally, the size of the largest connected component $N_{\mathrm{lcc}}$, the number of connected components $N_{\mathrm{cc}}$, and the global clustering coefficient *C* are used to quantify the level of investor category herding tendency. The larger the giant component, the more widespread the dominant behavior in the market. Similarly, the fewer components there in the network, the more homogeneous the trading strategies of different investor groups. The global clustering coefficient is calculated as the percentage of closed triplets from the total number of nodes’ triplets. A triplet is three nodes connected by either two (open triplet) or three (closed triplet) links:

(6) $$C=\frac{\sum_{i,j,k}A_{ij}A_{jk}A_{ki}}{\sum_{i}k_{i}\left(k_{i}-1\right)}.$$

Furthermore, we use the path-based measures such as average path length *l*, Wiener index *W*, and the average global efficiency *E* to capture the features of investor category networks:

(7) $$\begin{array}{cc} l & =\frac{1}{n\cdot (n-1)}\cdot \sum_{i\neq j}d\left(i,j\right), \end{array}$$

(8) $$\begin{array}{cc} W & =\frac{1}{2}\sum_{i\neq j}d\left(i,j\right), \end{array}$$

(9) $$\begin{array}{cc} E & =\frac{1}{n\cdot (n-1)}\cdot \sum_{i\neq j}\frac{1}{d(i,j)}, \end{array}$$

where d(i,j) is the length of the shortest path between nodes *i* and *j*. The average path length *l* is computed as the average length of all of the shortest paths, while the Wiener index *W* is defined as the sum of the shortest-path distances between each pair of nodes. The small size of the shortest path length indicates the emergence of investor hubs that make the average paths shorter. Hubs make networks better connected, thus shrinking the distances. Since the shortest path is defined as infinite if there is no path between two nodes, we calculate the average path length and the Wiener index only for the largest connected components where all nodes are reachable. The average global efficiency *E* is defined as the average of the inverse shortest path lengths between all node pairs (9). For node pairs that do not have a path between them, the distance equals infinity, and the inverse equals zero. For this reason, we can compute the global efficiency measure for all nodes in the network.

Finally, we leverage three types of node centrality measures to compare the network changes in terms of respective measure heterogeneity using the Gini coefficient as well as graph centrality indices based on them [25,26]. For each node *i* we use a degree centrality $k_{i}^{c}$, closeness centrality $c_{i}^{c}$ and betweeness centrality $b_{i}^{c}$ defined as follows:

(10) $$k_{i}^{c}=\frac{k_{i}}{N-1},$$

(11) $$c_{i}^{c}=\frac{N-1}{\sum_{i\neq j}d(i,j)},$$

(12) $$b_{i}^{c}=\sum_{s\neq v\neq t}\frac{\sigma_{st}\left(i\right)}{\sigma_{st}},$$

where $\sigma_{st}$ is the total number of shortest paths between nodes *s* and *t* and $\sigma_{st}\left(i\right)$ is the number of shortest paths between nodes *s* and *t*. For a given centrality measure *c* the network’s Gini coefficient is calculated as:

(13) $$G_{c}=\frac{\sum_{i,j}|c_{i}-c_{j}|}{2N\sum_{i}c_{i}}.$$

The Gini coefficient ranges between 0 and 1, where 0 indicates minimum and 1 indicates maximum heterogeneity in terms of the observed node centrality measures. The graph centrality index CI for a centrality measure *c* is defined as:

(14) $$CI_{c}=\frac{\sum_{i}\left(c^{*}-c_{i}\right)}{\max\left\{\sum_{i}\left(c^{*}-c_{i}\right)\right\}},$$

where $c^{*}=\max_{i}c_{i}$ is the maximum observed value of the centrality measure in the investigated network, while the denominator is calculated for the same size graph that provides the maximum value of the quantity. As it turns out the maximum values of the denominator for the investigated centrality measures are achieved for a star graph. CI ranges from 0 to 1, taking the value of 0 if the centralities of all nodes are equal, and a value of 1 if the network is a star tree. The graph centrality index based on degree centrality $CI_{c^{k}}$ was recently proposed as a measure of market centralization in a network of securities [27].

**Figure A1:**
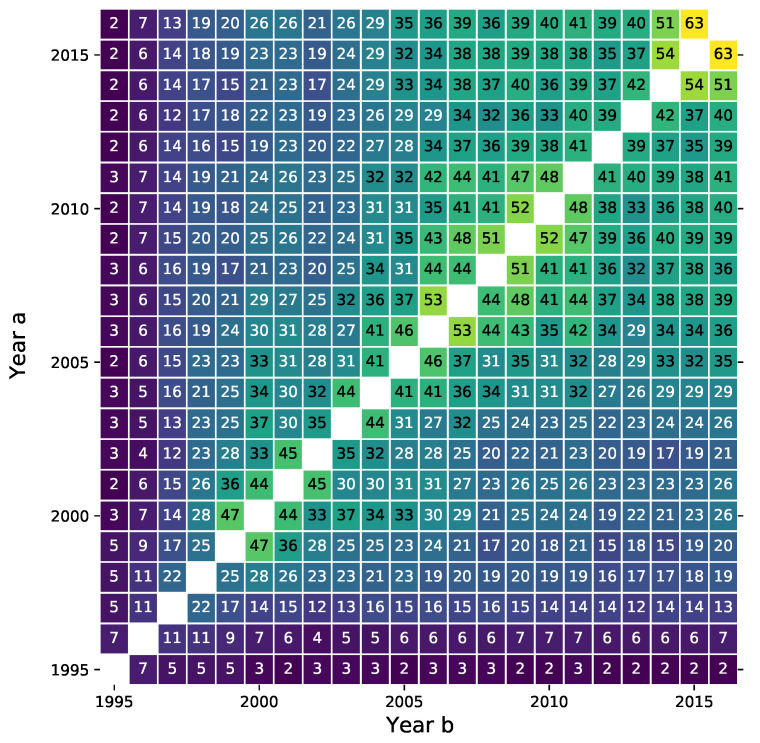
Number of common investor networks estimated for different pairs of periods.

Having 22 annual distributions composed of the features calculated for different security networks in a given year, we conduct a number of dependent *t*-tests for paired samples of network structure indicators (for the number of observations used in each test, see Figure A1 in Appendix A). In particular, we perform 231 (=22×21/2) tests for each of the 10 network features to determine if we can reject the null hypothesis of the two sets of annual network features having the same sample mean.

## 3. Results

**Figure 2:**
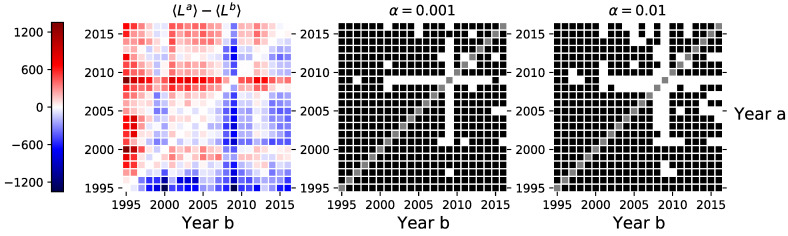
Left-hand side sub-figure shows the differences in average number of links *L* between investor category networks inferred in different periods. The central and right-hand side sub-figures indicate the pairs of years where we could not reject the null hypothesis of the dependent sample mean *t*-test (■), and the pairs of years where we rejected the null in favor of the alternative (□).

In this section, we present the results for all analyzed network features. In order for the results to be robust against the differences of investor behavior in different securities, for a given pair of periods, we compare network features only for the securities that had an inferred network in both of them (see Figure A1 for the number of observations). We provide two figures showing pairs of periods for each network feature for which we reject the null hypothesis of equal sample means at significance α equal to 0.001 and 0.01. In particular, the null and alternative hypotheses for the network feature *f* test are defined as follows:

(15) $$\begin{array}{cc} H_{0}: & \langle f^{a}\rangle =\langle f^{b}\rangle \; \end{array}$$

(16) $$\begin{array}{cc} H_{a}: & \langle f^{a}\rangle \neq \langle f^{b}\rangle , \end{array}$$

where $\langle f^{a}\rangle$ is the mean of a particular network feature *f* observed in year *a*. The ■ in the figures indicates for which pairs of periods we have failed to reject the null hypothesis (the means are equal), and the □ indicates for which pairs of periods we have rejected the null hypothesis in favor of the alternative (the means are different) with α∈{0.001,0.01} (e.g., see Figure 2). Moreover, each figure is accompanied by a heat map to visualize the differences between the sample means in different periods. The color of each cell encodes the difference between the mean value of a feature observed in the year indicated in the y-axis and the mean value of the year indicated in the x-axis.

### 3.1. Links, Density, and Average Degree

The left-hand side of Figure 2 shows the differences between the average number of links *L* observed between different years. The same heat map can represent the density ρ and average degree 〈k〉, though on a different scale (not shown in the figure). That is, all three measures are linearly dependent on the number of links in the network, yet scaled differently (see (3) to (5)), for which reason we report results only for the average number of links. From the figure, we can see that the network connectivity peaked in 2008–2009 with more links on average (higher density and average degree) than observed in other years. Moreover, when comparing the means of the annual network features that are calculated for the securities with existing networks in both compared periods, we can see that the years 2008–2009 particularly stand out as with only a few exceptions, we rejected the paired *t*-test null hypotheses between the ones observed in those years and the ones observed between 2001 and 2016.

From the point of view that the network estimation is based on the synchronization measures in the trade timing, we provide evidence that investors timed their transactions more similarly during the 2008–2009 crisis compared to other periods. This can result as a consequence of the use of similar trading strategies or a higher throughput in the investor information networks [5,29].

### 3.2. Global Clustering, Size of the Largest Connected Component, and the Average Number of Components

**Figure 3:**
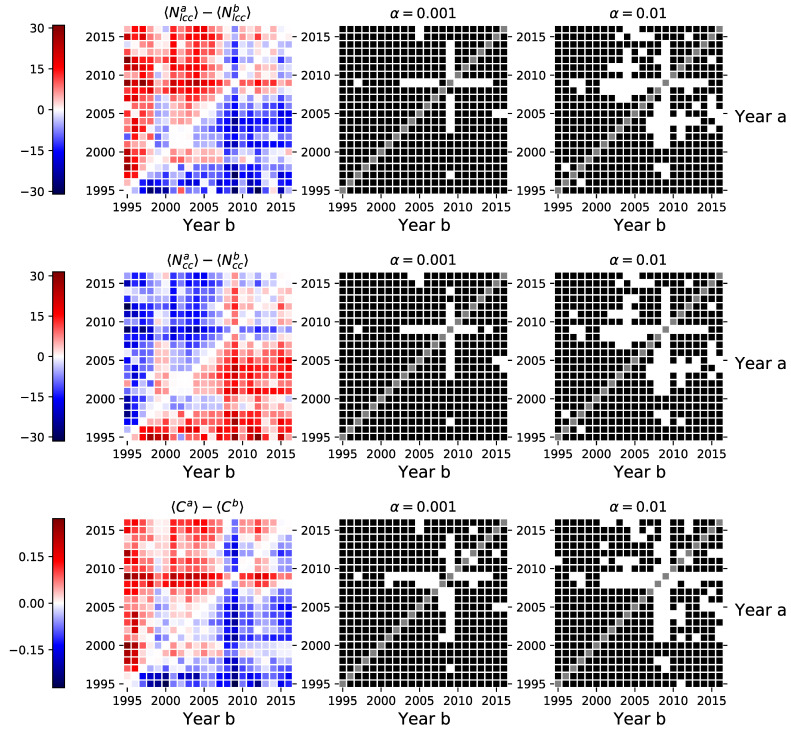
Left-hand side shows the differences in the annual mean values for the size of the largest connected component, number of components, and the global clustering coefficient between networks in different periods. The central and right-hand side sub-figures indicates the pairs of years where we could not reject the null hypothesis of the dependent sample mean *t*-test (■), and the pairs of years where we rejected the null in favor of the alternative (□).

Next, we compare the herding intensity between pairs of periods via three variables–the size of the largest connected component $N_{\mathrm{lcc}}$, the number of components in the network $N_{\mathrm{cc}}$, and global clustering coefficient *C*. The larger the giant component, the more the distinct investor categories use similar strategies, which results in a higher similarity in their trades timing. Similarly, the more the number of distinct components go down, the more there are investor categories that have synchronized trading patterns. The average clustering coefficient increases when the increased trading synchronization results in more closed node triplets, i.e., when two nodes sharing a common neighbor are also connected themselves. We can see from Figure 3 that global clustering and the size of the largest connected component are somewhat larger during the 2009 crisis, while the number of components in the networks is lower in 2009 than in other years. Again, the paired *t*-test mostly rejects the null hypothesis in favor of the alternative hypothesis where one of the network feature distributions comes from the crisis period in 2009. It means that the distributions of all three network features have different means during the crisis when compared to other periods.

### 3.3. Average Distance, Wiener Index, and Global Efficiency

**Figure 4:**
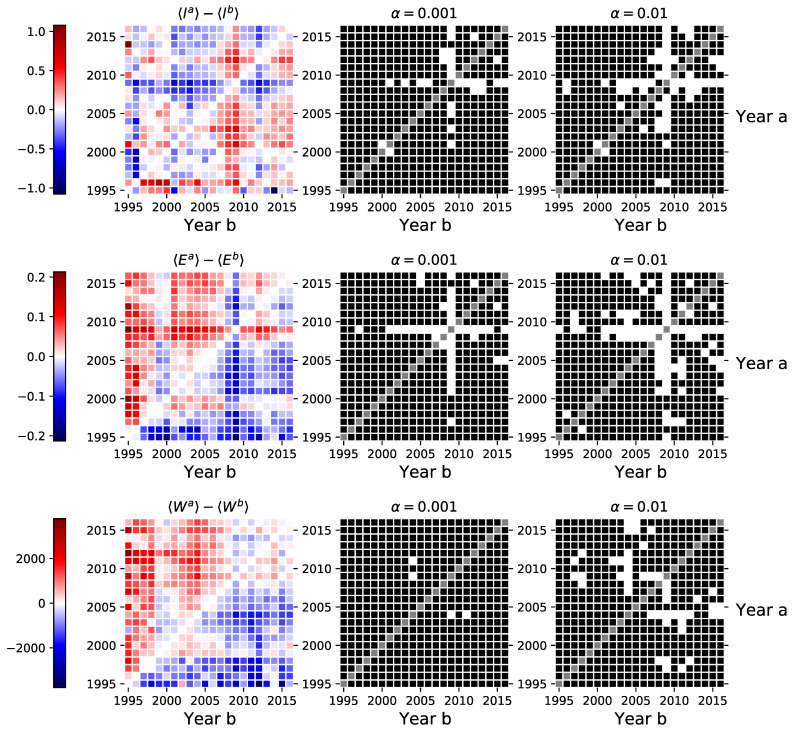
Left-hand side shows the differences in the annual mean values for the average shortest path length in the giant component, global network efficiency, and Wiener index for networks in different periods. The central and right-hand side sub-figures indicates the pairs of years where we could not reject the null hypothesis of the dependent sample mean *t*-test (■), and the pairs of years where we rejected the null in favor of the alternative (□).

Next, we turn our attention to the path-based network features. We calculate the average distance *l* (7) and the Wiener index *W* (8) for the largest connected components, while the global efficiency *E* (9) is calculated for whole networks. As we can see from Figure 4, the Wiener index increases with time with no apparent change during the financial crisis, i.e., $W^{a}>W^{b}$ for many *a* and *b*, when a>b (see the red color dominating above the diagonal of the matrix). On the other hand, in 2008–2009, the average distance is lower and the global efficiency higher compared to the other periods. This indicates that the nodes (investors) were more closely connected during the crisis. Since the Wiener index is defined as the sum of all shortest paths, the lack of sensitivity to the financial crisis in the Wiener index is explained by the increase in the number of links being offset by the decrease in the average path distances. During the crisis, investor hubs emerge, increasing the size of the largest connected components, shortening the distances between investor categories in the network, and making networks more connected.

### 3.4. Network Centrality

**Figure 5:**
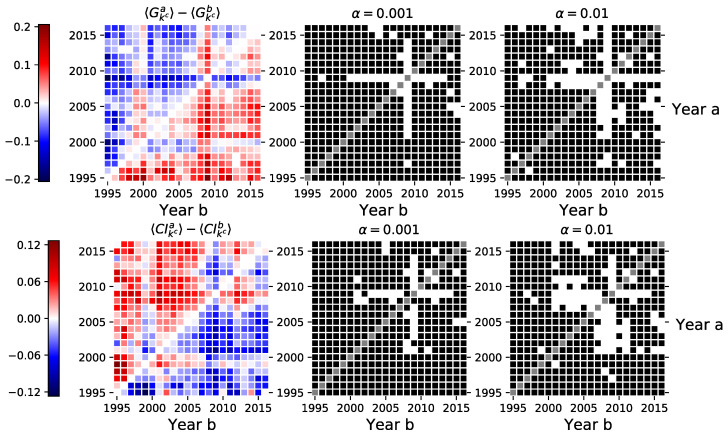
Left-hand side sub-figures show the differences in the Gini and graph centrality indices calculated using the degree centrality measure between investor category networks inferred in different periods (see Equations (10), (13) and (14)). The central and right-hand side sub-figures indicate the pairs of years where we could not reject the null hypothesis of the dependent sample mean *t*-test (■), and the pairs of years where we rejected the null in favor of the alternative (□). Similar figures for indices based on closeness and betweenness centralities are provided in the Appendix A.

Finally, we take a look at two different global network indices based on three different node centrality measures. Here we present only the indices based on the degree centrality in Equation (10). Indices based on the closeness and betweenness centralities are provided in the Appendix A. The absolute differences in the average coefficients range roughly up to 0.1, see Figure 5. The absolute differences for the average Gini coefficient range up to 0.2 and up to 0.12 for the average graph centrality index. Both of the figures suggest statistically significant structural changes in the investor networks during the financial crisis of 2008–2009. However, when these measures are calculated for the largest connected components in the corresponding networks, we can not make similar conclusions, see Figure A7 in the Appendix A.

### 3.5. Robustness Checks

**Figure A2:**
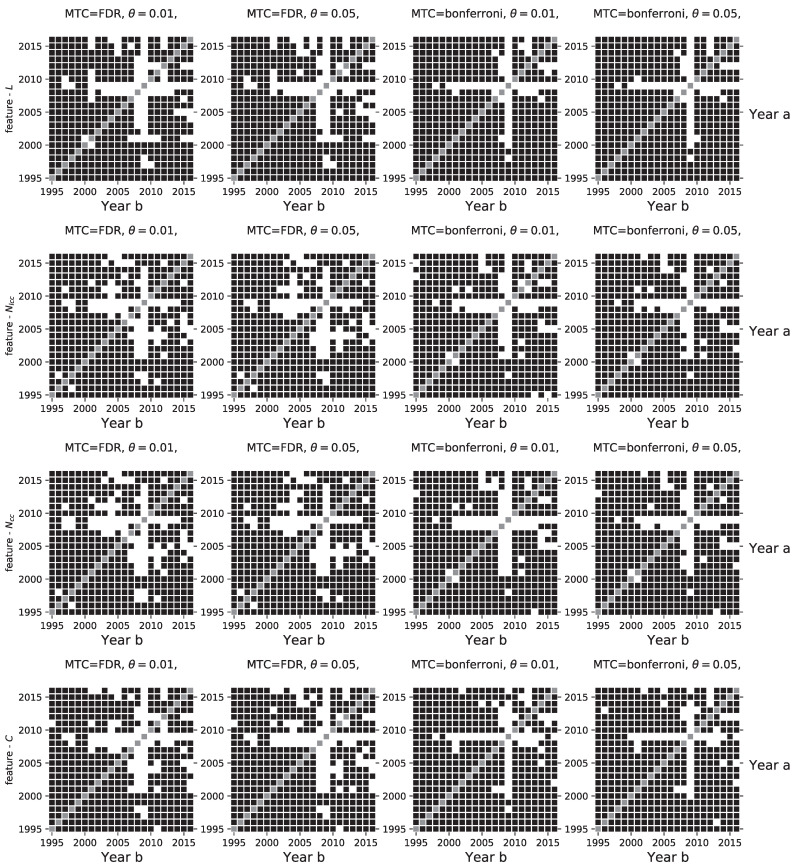
Figures indicate the pairs of years where we could not reject the null hypothesis of the dependent sample mean *t*-test (■), and the pairs of years where we rejected the null in favour of the alternative (□), at statistical significance α=0.01. Each row summarizes the results for different network features, namely the number of links *L*, the size of the largest connected component $N_{lcc}$, the number of connected components $N_{cc}$, and the average clustering coefficient *C*. Subplots in each row differ in the multiple correction applied (first two columns—FDR, last two—Bonferroni) and the θ parameter (first and third columns use θ=0.01, second and fourth use θ=0.05) used to assign trading states based on net-scaled volume (see Equation (1)). The figures in the second column are identical with the ones presented in the article and are shown here for easier comparison.

**Figure A3:**
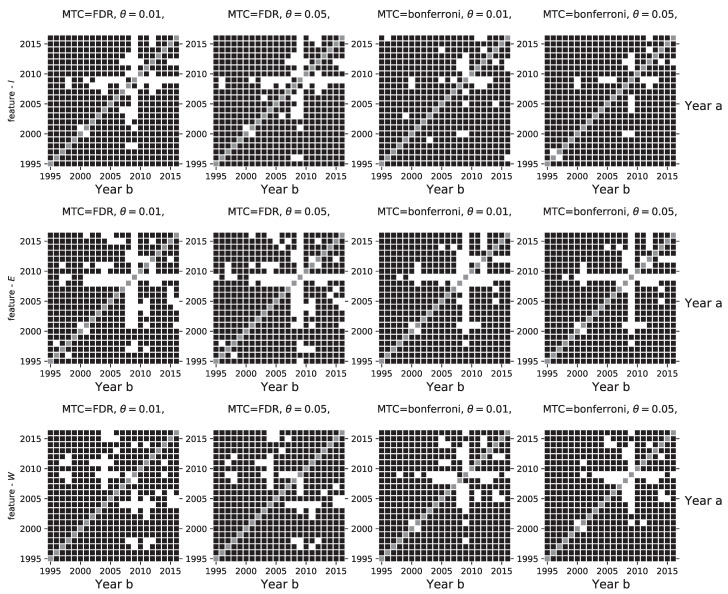
Figures indicate the pairs of years where we could not reject the null hypothesis of the dependent sample mean *t*-test (■), and the pairs of years where we rejected the null in favour of the alternative (□), at statistical significance α=0.01. Each row summarizes the results for different network features, namely the average shortest path length in the giant component *l*, the global network efficiency *E*, and the Wiener index *W*. Subplots in each row differ in the multiple correction applied (first two columns—FDR, last two—Bonferroni) and the θ parameter (first and third columns use θ=0.01, second and fourth use θ=0.05) used to assign trading states based on net-scaled volume (see Equation (1)). The figures in the second column are identical with the ones presented in the article and are shown here for easier comparison.

**Figure A7:**
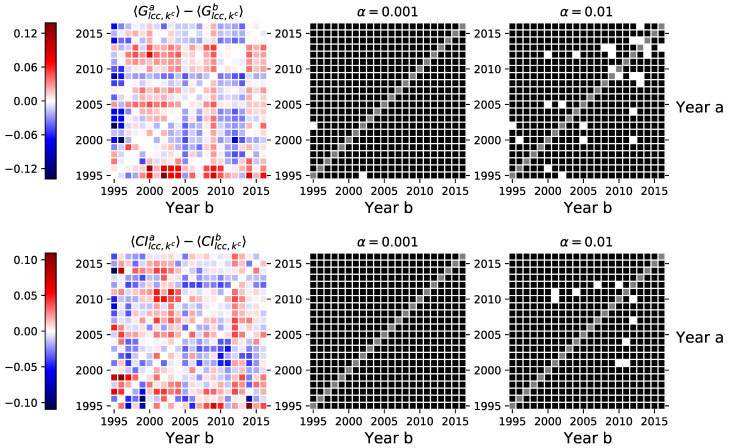
Left-hand side sub-figures show the differences in the Gini and graph centrality indices calculated using the degree centrality measure between the largest connected components in investor category networks inferred in different periods (see Equations (10), (13) and (14)). The central and right-hand-side sub-figures indicates the pairs of years where we could not reject the null hypothesis of the dependent sample mean *t*-test (■), and the pairs of years where we rejected the null in favour of the alternative (□). Similar figures result when Gini and graph centrality indices are calculated using the closeness and betweeness centrality measures. Figure are available upon request.

For a robustness check, we run the results with both FDR and Bonferroni multitest correction methods. Moreover, we vary the threshold parameter θ used in Equation (1), which determines the regions where a trading day is assigned into the buying or the selling states. Figure A2 and Figure A3 in the Appendix A show that the results are very consistent not only with respect to the multitest correction methods but also with respect to θ. In fact, with most of the features, the use of Bonferroni sharpens the contrast of 2008–2009 to other years, thus strengthening the results. The level of θ has a barely noticeable impact on the results.

## 4. Conclusions

How does the investor trading behavior and their mutual interactions in the stock market change during a financial crisis? In this paper, we answered this question by analyzing the structure of investor networks with some of the most prominent network features. The main empirical contribution of this paper is the finding that all of the investigated features were abnormally distributed during the 2008–2009 financial crisis period.

Our results are robust, showing significantly different distributions for 2008–2009 compared to the other 20 years on our sample. Most importantly, during the crisis, investors had abnormally many links to other investors, which indicates that investors were better linked and that the role of private information was more important compared to other times. Moreover, in terms of the network components and path-based network features, the results of the paper suggest an increased trading synchronization during the crisis. This further indicates the increased importance of private information channels during the crisis. On the other hand, the structure of investor networks did not show significant changes around the dot-com bubble. The finding that the investor networks reacted differently to different crises remains unexplained and requires further research.

The results of this paper are important for the further development of agent-based models. Notably, our results suggest that investors’ co-behavior is dependent on the state of the markets, which should be better captured by the models for agent behavior. Moreover, in future research, one could develop methods to reveal early-warning signals of financial crises. Such research has already been successfully reported in the context of inter-bank networks [16] and world trade [44]. We think this paper motivates such research in the context of investor networks, too.

## Figures and Tables

**Table 1:** Distribution of active investors across different sectors. The columns representing different investor sector types are as follows: European Union institutions (EU), financial and insurance corporations (F.&I.), government institutions (Gov.), households (House.), non European Union institutions (n.EU), non-financial corporations (n.F.), non-profit institutions (n.Prof.), and foreigner accounts (For.).

	EU	F.&I.	Gov.	House.	n.EU	n.F.	n.Prof.	For.	Total
1995	74	214	106	52,356	6	4565	667	12	58,000
1996	51	287	118	63,425	6	4816	774	27	69,504
1997	68	340	122	91,306	5	6395	987	35	99,258
1998	90	339	129	129,276	3	8043	1213	69	139,162
1999	95	440	148	182,874	3	10,425	1556	368	195,909
2000	336	528	178	272,843	29	14,368	1664	509	290,455
2001	728	417	139	200,442	179	10,455	1382	760	214,502
2002	641	409	147	144,504	216	8205	1229	693	156,044
2003	997	456	139	147,467	466	8439	1198	539	159,701
2004	998	441	290	163,798	603	8822	1413	613	176,978
2005	1031	608	132	182,679	390	9445	1382	222	195,889
2006	1084	445	179	176,352	424	9768	1359	423	190,034
2007	1412	421	116	194,991	964	9948	1398	702	209,952
2008	629	402	94	153,957	216	8818	1010	598	165,724
2009	536	392	128	194,298	181	9635	1667	312	207,149
2010	830	527	108	217,414	406	12,117	1432	630	233,464
2011	981	532	100	247,829	558	12,489	1470	600	264,559
2012	1292	515	109	230,791	1128	11,397	1415	553	247,200
2013	2025	509	124	267,791	1996	12,534	1613	512	287,104
2014	2051	526	131	279,515	1894	12,901	1823	330	299,171
2015	2100	540	158	267,429	2327	12,849	1761	478	287,642
2016	2169	514	131	266,175	2535	12,473	1575	450	286,022

**Table 2:** Average network node statistics in a given year. ’avg. inv. tr.’ indicates the average number of trades executed by an investor belonging to a given investor category. ’n. inv.’ indicates the average number of investors in a given category. ’n. trades’ indicates the average number of trades by a given investor category. ’n. sec.’ indicates the average number of securities investors in a given category have traded. ’n. inv. sec.’ indicates the average number of securities an investor trades in a given category.

	avg. inv. tr.	n. inv.	n. trades	n. sec.	n. inv. sec.
1995	42.15	542.06	3117.70	66.70	3.75
1996	38.44	637.65	4321.51	78.32	4.23
1997	44.67	919.06	6370.85	87.13	4.15
1998	35.47	1276.72	8454.65	97.32	4.17
1999	22.09	1797.33	13,043.98	116.59	4.47
2000	21.19	2593.35	21,874.32	124.41	4.43
2001	24.16	1932.45	16,758.54	136.21	4.39
2002	24.17	1431.60	13,944.69	160.52	4.47
2003	22.20	1478.71	14,091.56	172.62	4.70
2004	21.78	1608.89	16,226.59	202.10	4.51
2005	29.90	1749.01	19,354.29	238.31	5.38
2006	30.56	1727.58	22,532.95	318.73	5.41
2007	30.42	1874.57	24,952.73	372.95	5.42
2008	31.38	1506.58	24,809.50	354.93	5.36
2009	25.59	1866.21	26,793.68	280.45	5.10
2010	36.27	2122.40	33,096.51	394.26	6.21
2011	37.32	2405.08	32,940.02	512.27	6.06
2012	30.82	2332.08	29,387.09	534.23	5.48
2013	32.55	2708.53	33,650.18	617.48	5.94
2014	37.09	2719.74	33,143.83	712.45	5.90
2015	35.27	2688.24	36,139.37	886.69	6.25
2016	37.06	2624.06	36,650.42	758.92	6.28

**Figure A4:**
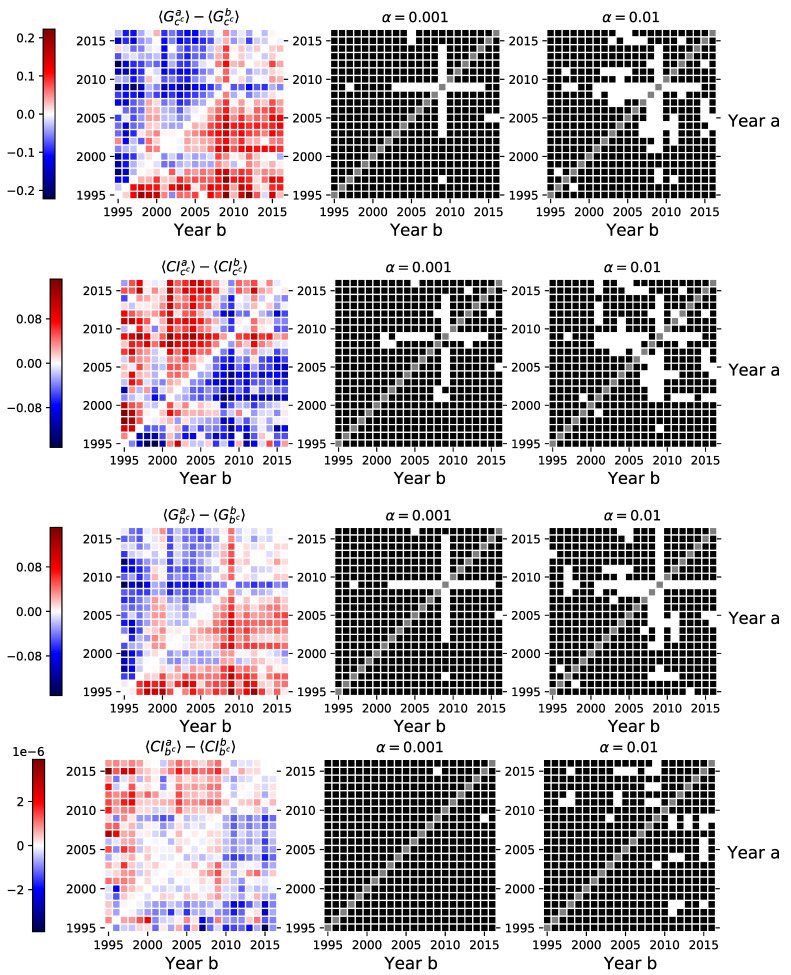
Left-hand side sub-figures show the differences in the Gini and graph centrality indices calculated using the closeness and betweeness centrality measures between investor category networks inferred in different periods (see Equations (11)–(14)). The central and right-hand-side sub-figures indicates the pairs of years where we could not reject the null hypothesis of the dependent sample mean *t*-test (■), and the pairs of years where we rejected the null in favor of the alternative (□). Similar figures for indices based on closeness and betweenness centralities are provided in the Appendix A.

**Figure A5:**
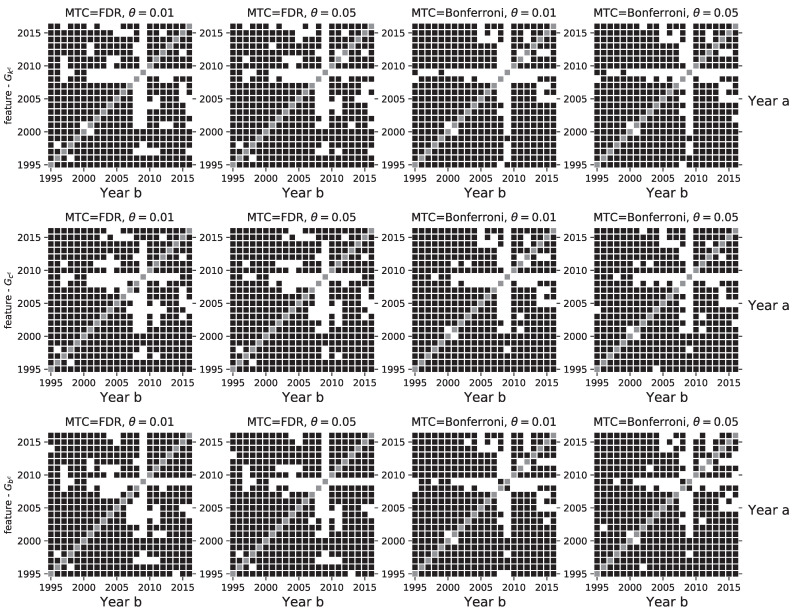
Figures indicate the pairs of years where we could not reject the null hypothesis of the dependent sample mean *t*-test (■), and the pairs of years where we rejected the null in favor of the alternative (□), at statistical significance α=0.01. Each row summarizes the results for Gini coefficients calculated using different node centrality measures, namely the degree centrality $k^{c}$, closeness centrality $c^{c}$, and the betweeness centrality $b^{c}$. Subplots in each row differ in the multiple correction applied (first two columns—FDR, last two—Bonferroni) and the θ parameter (first and third columns use θ=0.01, second and fourth use θ=0.05) used to assign trading states based on net-scaled volume (see Equation (1)). The figures in the second column of the first row are identical with the one presented in the article and is shown here for easier comparison.

**Figure A6:**
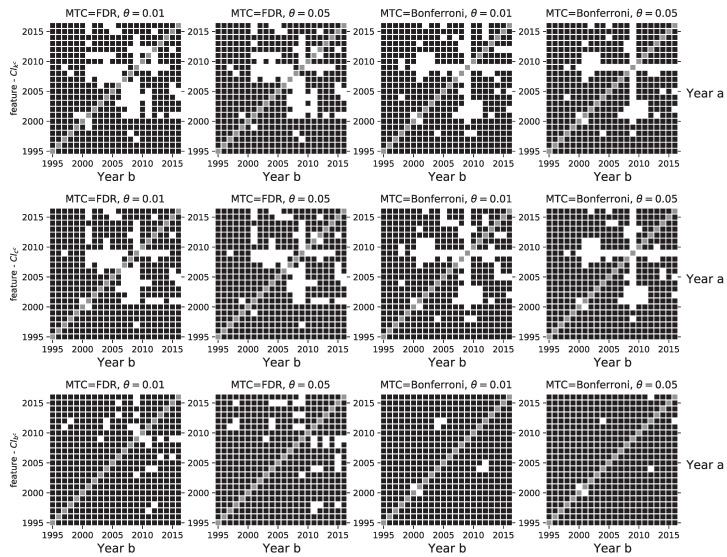
Figures indicate the pairs of years where we could not reject the null hypothesis of the dependent sample mean *t*-test (■), and the pairs of years where we rejected the null in favor of the alternative (□), at statistical significance α=0.01. Each row summarizes the results for the graph centrality indices calculated using different node centrality measures, namely the degree centrality $k^{c}$, closeness centrality $c^{c}$, and the betweeness centrality $b^{c}$. Subplots in each row differ in the multiple correction applied (first two columns—FDR, last two—Bonferroni) and the θ parameter (first and third columns use θ=0.01, second and fourth use θ=0.05) used to assign trading states based on net-scaled volume (see Equation (1)). The figures in the second column of the first row are identical with the one presented in the article and is shown here for easier comparison.

## Data Availability

Restrictions apply to the availability of these data. Data was obtained from Euroclear Finland Oy and are available from the data provider under a non-disclosure agreement.

## Appendix A. Descriptive Statistics

Table A1 summarizes the numbers of security-specific networks that were inferred in different years. The columns in the table present the number of networks resulting after different link validation decisions, i.e., no validation, when each link is validated with statistical significance α=0.05, when FDR multiple test correction is applied, and when the largest connected component is required to have more than 5 nodes.

**Table A1:** Number of networks observed in different years. The columns summarize the number of security-networks that (i) have at least one non-validated link, (ii) have at least one statistically significant link with a *p*-value lower than 0.05 verified without multiple test correction (MTC), (iii) have at least one link remaining after the false discovery rate (FDR) for the MTC, and (iv) had more than 5 nodes in the largest connected component after the FDR for MTC was applied. These numbers are used to produce Figure 1.

	Securities	No MTC	FDR MTC	FDR MTC ($N_{\mathrm{lcc}}>5$)
1995	128	126	38	12
1996	135	134	62	27
1997	142	139	74	37
1998	166	163	92	55
1999	203	197	108	70
2000	225	213	118	89
2001	350	309	125	81
2002	527	453	160	89
2003	672	506	184	96
2004	885	667	244	138
2005	1137	851	298	161
2006	1503	1191	463	251
2007	2139	1663	501	230
2008	2126	1646	469	228
2009	1474	1133	421	230
2010	2456	1682	394	185
2011	4578	2617	424	174
2012	5682	2840	335	107
2013	5388	2980	460	178
2014	6747	3679	399	149
2015	10,086	4734	436	165
2016	11,090	4320	421	167
